# The relationship between the presence of anti-cyclic citrullinated peptide antibodies and clinical phenotype in very early rheumatoid arthritis

**DOI:** 10.1186/1471-2474-11-187

**Published:** 2010-08-23

**Authors:** Mohammed Z Cader, Andrew D Filer, Christopher D Buckley, Karim Raza

**Affiliations:** 1 MRC Centre for Immune Regulation, School of Immunity and Infection, The University of Birmingham, Birmingham B15 2TT, UK; 2 Department of Rheumatology, Sandwell and West Birmingham Hospitals NHS Trust, Birmingham B18 7QH, UK

## Abstract

**Background:**

Anti-cyclic citrullinated peptide (anti-CCP) antibodies are highly specific for RA, but are not detectable in all RA patients. The aim of this study was to establish whether the clinical phenotypes of anti-CCP positive and negative disease are distinct at the earliest clinically apparent phase of disease.

**Methods:**

Patients were recruited from the Birmingham early inflammatory arthritis clinic. Participants were included in the current study if they presented within 3 months of symptom onset and fulfilled 1987 ACR criteria for RA at some point during an 18 month follow-up. Data were collected on demographic variables, joint symptoms and tender (n = 68) and swollen (n = 66) joint counts. CRP, ESR, rheumatoid factor and anti-CCP2 status were measured.

**Results:**

92 patients were included (48 anti-CCP positive). The anti-CCP positive and negative groups were comparable in terms of demographic variables, inflammatory markers, joint counts and 1987 ACR classification criteria, except that more anti-CCP positive patients were rheumatoid factor positive (83.3% vs. 11.4%, p < 0.01). There was no significant difference in the pattern of joint involvement, except for an increased prevalence of knee joint swelling in anti-CCP positive patients (42.9% vs. 22.2%, p = 0.03).

**Conclusions:**

Patients with and without anti-CCP antibodies present in a similar way, even within three months of clinically apparent disease that eventually develops into RA.

## Background

Rheumatoid arthritis (RA) is a chronic, inflammatory condition typically manifesting clinically as a symmetrical polyarthritis. Rheumatoid synovitis is characterised by complex leukocyte and cytokine networks. The persistence of inflammation is mediated, in part, by the stromal micro-environment, but the underlying causes remain unclear [1,2]. Over the last decade there has been particular interest in antibodies to citrullinated peptides and proteins as important aetiological and predictive factors in early RA [3-5]. Citrullination of proteins is a post-translational modification, which can occur as a normal part of cell apoptosis [6]. However, this process may induce antibody formation in susceptible individuals [7], which may predate clinical arthritis by several years [8]. Subsequent environmental triggers may enable anti-citrullinated protein/peptide antibodies to enter joints and contribute to a chronic inflammatory response [9].

Anti-cyclic citrullinated peptide (anti-CCP) antibodies are highly specific for RA, but are not detectable in all patients [10]. This raises the possibility that distinct mechanisms exist for the pathogenesis of synovitis in anti-CCP positive and negative patients. Indeed, anti-CCP positive patients show both environmental and genetic associations not present in anti-CCP negative RA. For example, tobacco smoking is a well-recognised risk factor for anti-CCP positive RA especially amongst HLA-DRB1 individuals expressing the 'shared epitope' [11]. Furthermore anti-CCP positive patients have more severe radiological destruction and poorer outcomes [12], and synovial pathology appears to differ according to anti-CCP status in the established phase of RA [13].

A recent study of RA patients presenting within 2 years of symptom onset, suggested no clinical phenotypic differences according to anti-CCP status [12]. However, it is possible that as the disease evolves, all RA patients, regardless of anti-CCP status, develop a common pattern of joint involvement and that differences were not observed because the symptom duration at inclusion was too heterogeneous. Moreover, there is evidence that pathogenic mechanisms in the first few months may differ from those in longer duration disease and that this phase may be more responsive to therapy [14,15]. Hence we aimed to establish whether the clinical phenotypes of anti-CCP positive and negative disease were distinct at the earliest clinically apparent phase of RA, within 3 months of symptom onset.

## Methods

Patients were recruited from the rapid access early inflammatory arthritis clinic at Sandwell and West Birmingham Hospitals NHS Trust. Patients referred to the clinic by their General Practitioners were seen within 2 weeks. Participants were included in the current study if they presented within 3 months of the onset of any symptom attributed by the assessing Rheumatologist to inflammatory joint disease (pain, stiffness, swelling), had clinically apparent synovial swelling at baseline and fulfilled 1987 American College of Rheumatology criteria (ACR) for RA, either at baseline or during 18 months follow-up [16].

Data were collected on patient demographic variables, fulfillment of the ACR criteria, duration of symptoms and whether the mode of onset was acute or insidious. Tender (n = 68) and swollen (n = 66) joint counts were performed. CRP, ESR, rheumatoid factor and anti-CCP2 status were measured at baseline. Radiographs were performed of the hands and feet. Systematic clinical follow-up was carried out at 1, 2, 3, 6, 12 and 18 months.

The anti-CCP positive and negative groups were compared, with differences in means assessed using a two-tailed unpaired student t-test. Proportions were compared using a chi-squared test. Data analysis was performed using the Statistical Package for Social Sciences, version 17.0 (SPSS Institute, Chicago, IL, USA). P values < 0.05 were considered significant. Patients gave their informed consent prior to inclusion into the programme. The study received ethical approval from the local research ethics committee.

## Results

At the time of this analysis 265 patients with an initial symptom duration of less than 3 months and with completed follow-up had been recruited to the early synovitis cohort; of these, 92 patients (34.7%) fulfilled 1987 ACR criteria for RA at some point during follow-up and were included in the present study. Of these 92 patients, 48 patients had anti-CCP antibodies and 44 patients had no anti-CCP antibodies at inclusion. There were an additional 35 patients (13.2%) with persistent unclassified arthritis at the end of 18 month follow-up, all of whom were anti-CCP negative. 21 of these patients were treated with disease modifying anti-rheumatic drugs (DMARDs); if such agents had not been used, it is possible that they may have eventually reached a diagnosis of RA.

**Figure 1:**
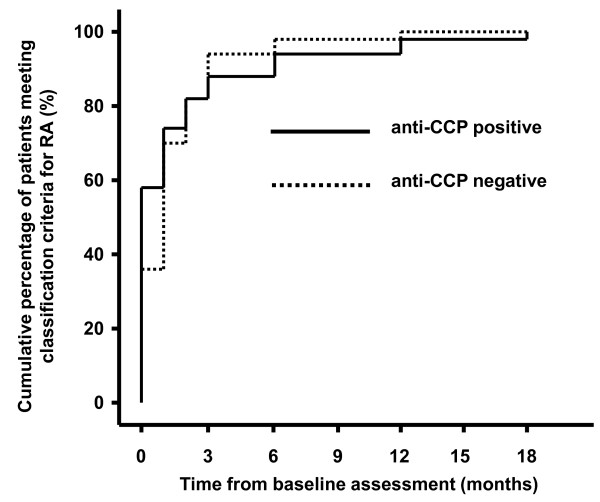
**Cumulative proportion of patients fulfilling 1987 ACR classification criteria for RA over 18 month follow-up period**. Solid line = anti-CCP antibody positive patients. Dashed line = anti-CCP antibody negative patients.

The patient groups were comparable in terms of gender, age, ethnicity and smoking status (Table 1). In addition, patients had similar durations of symptoms and modes of onset. There were no significant differences in the presence or duration of morning stiffness. Moreover, the other 1987 ACR criteria for RA were equally likely to be present in the anti-CCP positive and negative groups. This includes the presence of nodules and radiological evidence of erosions, both of which were very rare at presentation. The exception, however, was that anti-CCP positive patients were significantly more likely to be seropositive for rheumatoid factor (83.3% vs. 11.4%, p < 0.001). The mean number of ACR criteria present at baseline was greater for anti-CCP positive (3.73) than for anti-CCP negative patients (3.23) (p = 0.03). However, this was not significant after repeating the analysis with rheumatoid factor status excluded. There was also a significant difference in the proportion of patients meeting at least 4 criteria needed for a diagnosis of RA at baseline (anti-CCP positive, 58.3%; anti-CCP negative, 36.4%; p = 0.04). However, there was no difference in the mean time from symptom onset to the fulfillment of classification criteria for RA (anti-CCP positive, 158 days; anti-CCP negative, 131 days) in those who did not fulfill classification criteria at baseline (Figure 1). It should be noted that the 1987 ACR criteria stipulate that articular symptoms and signs should have been present for at least 6 weeks; this criterion was included in the present analysis. 28 patients who eventually fulfilled 1987 ACR criteria for RA (15 anti-CCP positive, 13 anti-CCP negative) were seen in clinic within 6 weeks of the onset of symptoms and hence, by definition, were unable to fulfill 1987 ACR critera for RA at baseline. Of these, 15 patients (8 anti-CCP positive, 7 anti-CCP negative) would have met sufficient criteria needed for diagnosis at baseline, had the 6 week criterion been disregarded.

**Table 1:** Characteristics of patients with and without anti-CCP antibodie

	anti-CCP positive (n = 48)	anti-CCP negative (n = 44)	P value
Female (n (%))	28 (58.3%)	22 (50.0%)	0.42*

Age at inclusion, years (mean ± standard deviation)	56.6 ± 15.0	61.5 ± 14.7	0.12**

Ethnicity - Caucasian (n (%))	38 (79.2%)	39 (88.6%)	0.22*

Smoking status - Current/ever smoked (n (%))	31 (64.6%)	28 (63.6%)	0.92*

Symptom duration, days (mean ± standard deviation)	49.3 ± 20.2	56.4 ± 22.3	0.12**

Mode of onset (n (%))			
Acute	25 (52.1%)	25 (56.8%)	0.65*
Insidious	21 (43.8%)	18 (40.9%)	
Unspecified	2 (4.16%)	1 (2.27%)	

Morning Stiffness			
Duration, mins (mean ± standard deviation)	93.3 ± 89.5	123.8 ± 92.8	0.11**
No. patients ≥60 mins (n (%))	28 (58.3%)	32 (72.7%)	0.15*

Symmetry (n (%))	35 (72.9%)	33 (75.0%)	0.82*

Hand joint involvement (n (%))	43 (89.6%)	41 (93.2%)	0.54*

≥ 3 joint areas involved (n (%))	31 (64.6%)	30 (68.2%)	0.63*

Nodules (n (%))	2 (4.17%)	1 (2.27%)	0.61*

Erosions (n (%))	1 (2.08%)	2 (4.54%)	0.51*

Rheumatoid factor positive (n (%))	40 (83.3%)	5 (11.4%)	< 0.001*

1987 ACR criteria for RA			
Total No. (mean ± standard deviation)	3.73 ± 1.09	3.23 ± 1.14	0.03**
No. fulfilling ≥ 4 criteria (n (%))	28 (58.3%)	16 (36.4%)	0.04*

Tender joint count (max 68) (mean ± standard deviation)	8.33 ± 7.80	9.66 ± 8.90	0.45**

Swollen joint count (max 66) (mean ± standard deviation)	5.79 ± 4.51	7.07 ± 5.83	0.25**

ESR mm/hr (mean ± standard deviation)	32.3 ± 20.1	38.7 ± 29.3	0.23**

CRP mg/L (mean ± standard deviation)	29.0 ± 27.0	31.4 ± 30.8	0.70**

Statistical test applied: *Chi-squared test, **Unpaired student t-test

**Figure 2:**
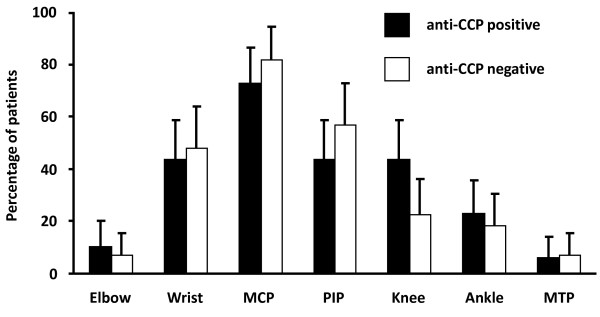
**Clinically apparent synovial swelling in individuals with and without anti-CCP antibody**. Joint involvement expressed as a percentage of the patients seen in clinic, with 95% confidence intervals. Black bars = anti-CCP antibody positive patients. White bars = anti-CCP antibody negative patients. MCP = metacarpophalangeal; PIP = proximal interphalangeal; MTP = metatarsophalangeal.

There were no significant differences in the total tender or swollen joint counts, number of affected joint areas or 28-joint score (as used in the DAS28 scoring for RA, data not shown). In addition, inflammatory markers were comparable in both groups. Amongst different joint regions, a significantly different prevalence of swelling was only seen in the knee joint, with a higher prevalence in anti-CCP positive patients (43.8% vs. 22.8%, p = 0.03) (Figure 2).

## Discussion

This study shows that RA patients with and without anti-CCP antibodies present similarly, even within three months of symptom onset. This is perhaps surprising given the emerging consensus that anti-CCP positive and negative states represent distinct clinical entities. There is mounting evidence suggesting not only separate molecular mechanisms underlying these two patient groups, but also different genetic and environmental predispositions as well as a different clinical progression. However, consistent with a previous study of patients presenting within 2 years of onset, our data provide evidence that, despite these pathological differences, there is a shared clinical phenotype for RA presentation regardless of anti-CCP status. Not only do patients with anti-CCP positive and negative disease present with similar distributions of joint disease, but they also have comparable ages of onset and levels of inflammation. However, anti-CCP positive patients do fulfill significantly more ACR criteria for RA than anti-CCP negative patients. This is predominantly due to the fact that anti-CCP positive patients more often express rheumatoid factor, a well described phenomenon [17]. Consequently, anti-CCP positive patients were more likely to have met at least 4 criteria, thereby fulfilling classification criteria for RA at initial presentation. However there were no significant differences in the proportions of patients meeting each of the other six 1987 ACR criteria when then anti-CCP positive and negative patients were compared.

This study has a number of limitations. Firstly, data are available to suggest that seropositivity for anti-CCP and rheumatoid factor are associated with delayed presentation in patients with RA [18,19]. These surprising observations raise the possibility that a subpopulation of seropositive individuals were not seen early enough for inclusion in our patient cohort. This may have introduced selection bias. Secondly, the study may have been underpowered to detect more subtle clinical differences between patient groups. A retrospective power analysis was carried out for each of the variables tested. This showed that the study had an 80% power to detect a difference in proportions between anti-CCP positive and negative groups of between 0.27 and 0.30 (27 to 30%) depending upon the particular joint area under consideration.

Interestingly, this study showed an increased likelihood of knee involvement at presentation in anti-CCP positive patients. This finding is consistent with other results of previous studies which have shown that anti-CCP positivity is associated with increased progression of radiological destruction and that initial knee involvement is also a positive predictor of greater radiological damage [20]. Our data suggest that the previously reported adverse outcomes of patients presenting with knee involvement may be due to the increased probability of anti-CCP seropositivity in such patients.

## Conclusions

In summary, anti-CCP positive and negative patients have similar baseline clinical presentations. This does not necessarily conflict with current opinion that antibodies to citrullinated peptides play an essential role in the pathogenesis of RA. The role of anti-CCP status in predicting progression of undifferentiated early arthritis into RA is well established [21,22], as is the association with increased bone destruction. It is possible that the shared clinical phenotype reported here may conceal differences that are occurring at a pathological level. This highlights the need to further investigate histological changes in early RA, which may demonstrate differences in anti-CCP positive and negative patients and have important therapeutic implications.

## Competing interests

KR, AF and CB hold unrestricted research grants from Wyeth, UCB and Cellzome. This study was supported by the European Community's Sixth Framework Programme (AutoCure).

## Authors' contributions

All authors were involved in study design and in drafting and revision of the manuscript. MZC, AF and KR performed the data collection, analysis and interpretation of the results. All authors have read and approved the final manuscript.

## Authors' Information

MZC: MA, MB BChir, Academic Foundation Year 2 Doctor in Rheumatology; ADF: PhD MRCP, Senior Lecturer in Rheumatology; CDB: PhD FRCP, Arthtitis Research UK Professor of Rheumatology; KR: PhD FRCP, Senior Lecturer in Rheumatology.

## Pre-publication history

The pre-publication history for this paper can be accessed here:

http://www.biomedcentral.com/1471-2474/11/187/prepub
